# Puerarin inhibits *Staphylococcus aureus*‐induced endometritis through attenuating inflammation and ferroptosis via regulating the P2X7/NLRP3 signalling pathway

**DOI:** 10.1111/jcmm.18550

**Published:** 2024-07-23

**Authors:** Chongshan Yuan, Li Liu, Ye Zhao, Ke Wang

**Affiliations:** ^1^ Department of Obstetrics China‐Japan Union Hospital of Jilin University Changchun Jilin China; ^2^ Department of Clinical Veterinary Medicine, College of Veterinary Medicine Jilin University Changchun Jilin China; ^3^ Reproductive Medicine Center China‐Japan Union Hospital of Jilin University Changchun Jilin China; ^4^ Department of Dermatological China‐Japan Union Hospital of Jilin University Changchun Jilin China

**Keywords:** endometritis, ferroptosis, NLRP3, P2X7, puerarin

## Abstract

Endometritis is one of the important causes of infertility. Puerarin (PU) can inhibit oxidative stress and reduce inflammation; however, it is unclear whether PU has a protective effect on the endometritis. In our study, we used *Staphylococcus aureus* to induce mouse endometritis. The PU group (100 mg/kg PU) and the *S. aureus* + PU group received daily intraperitoneal injection of PU (25, 50 or 100 mg/kg PU). The results showed that *S. aureus* significantly increased the levels of MPO, TNF‐α, IL‐1β and IL‐6 in uterine tissue, and increased the expression of p‐p65 and p‐IκBα proteins in uterine tissue to induce endometritis in mice (*p* < 0.05). Furthermore, it has been found that *S. aureus* promotes the occurrence of ferroptosis by reducing GSH and ATP content, increasing MDA and iron content and reducing GPX4 and SLC7A11 protein expression levels (*p* < 0.05). *S. aureus* significantly increase the expression of NLRP3, ASC, caspase‐1 and P2X7 proteins in uterine tissue (*p* < 0.05). However, PU obviously reduced the inflammatory response and reversed the changes of ferroptosis and the expression of P2X7 receptor/NLRP3 pathway associated proteins of the uterus induced by *S. aureus* (*p* < 0.05). Taken together, these findings emphasize the protective effect of PU on endometritis by regulating the P2X7 receptor/NLRP3 signalling pathway and inhibiting ferroptosis.

## 1 INTRODUCTION

Endometritis is an inflammatory disease caused by pathogenic microorganisms such as *Escherichia coli*, *Mycobacterium pyogenes* and *Staphylococcus aureus*, and is one of the important causes of infertility.^1^, ^2^ In addition, endometritis can cause a delay in the reproductive cycle, thereby reducing the production capacity of livestock and ultimately causing significant economic losses to farms.^3^ Postpartum and postmating are the high incidence periods of endometritis, both of which create a suboptimal environment for sperm motility, embryo development and placentation.^4^ Endometritis may reduce post‐partum animal milk production and maternal behaviour, which has a significant harm to the health and survival of offspring.^5^ More and more evidence suggested that targeted ferroptosis has great potential in the treatment of inflammatory diseases.^6^ Under normal conditions, iron levels are strictly regulated to maintain homeostasis, which can lead to ferroptosis when iron overload occurs.^7^ Ferroptosis is a novel form of nonapoptotic cell death driven by iron‐dependent lipid peroxidation, influenced by signalling pathways related to redox homeostasis, iron processing and inflammatory diseases.^8^ Ferroptosis can promote the release of inflammatory cytokines, which are involved in many inflammatory‐related disease processes.^9^ Recent studies have found that pulmonary artery endothelial ferroptosis triggers inflammatory responses through NLRP3 inflammasomes in rats.^10^ Inflammasomes are multi protein complexes in the cytoplasm responsible for the formation of pro‐inflammatory molecules. Among them, NLRP3 inflammasomes are associated with the progress of various diseases, including metabolic disorders, inflammatory bowel disease and other inflammatory diseases.^11^ In addition, NLRP3 is also involved in other chronic inflammatory diseases, such as gout or atherosclerosis.^12^ As a potent activator of NLRP3 inflammasome, P2X7 receptor is highly expressed in immune cells and is important to regulate inflammation.^13^ In addition, P2X7 is important for regulating both innate and adaptive immune responses in congenital bone marrow cells by inducing NLRP3 activation.^14^ P2X7 initiates the inflammatory response by activating NLRP3 inflammasome and releasing pro‐inflammatory cytokines IL‐1β and IL‐18.^15^ Puerarin (PU), as an isoflavone compound, is the main active ingredient in the traditional Chinese medicine Pueraria lobata.^16^ PU has anti‐inflammatory and antioxidant effects, and has been used to prevent and treat cardiovascular diseases.^17^ Recent studies have shown that PU can inhibit oxidative stress and reduce inflammation, alleviating myocardial injury caused by lipopolysaccharide (LPS).^18^ Furthermore, PU can improve LPS‐induced organ injury by inhibiting TLR4/NF‐κB/JNK signalling pathway.^19^ In vitro experiment, PU can reduce myocardial injury by inhibiting the expression of inflammatory cytokines such as IL‐1β and TNF‐α.^20^ Studies have shown that PU can alleviate inflammation by reducing ferroptosis, while reports of ferroptosis leading to endometritis are gradually increasing.^21^, ^22^, ^23^ However, whether PU reduces ferroptosis through the P2X7 pathway and thus has a protective effect on the inflammatory response of endometritis is not clear. Therefore, our study investigated the therapeutic effect of PU on endometritis and explore its mechanism, which can open up a new treatment option for exploring the use of PU in the treatment of endometritis.

## 2 MATERIALS AND METHODS

### 2.1 Animals

The BALB/c mice were purchased from the Center of Experimental Animals of Baiqiuen Medical College of Jilin University. Select female mice aged 8–10 weeks, weighing 25–30 g and raise them at room temperature without pathogenic bacteria, the mice were provided tap water and food ad libitum. After acclimation for 10 days, the mice were randomly divided into six groups containing 12 mice each. All operations were carried out in accordance with the Guidelines for Care and Use of Laboratory Animals of Jilin University requirements and were approved by the Animal Ethics Committee of Jilin University.

### 2.2 Materials

*S. aureus* SA113 (ATCC35556) was purchased from Mikrobielle Genetik, University of Tubingen, Germany and cultured in Mueller‐Hinton II cation‐adjusted broth (MH, BD Biosciences, Sparks, MD, USA) at 37°C. Mouse monoclonal antibodies GPX, SLC7A11, NF‐κB p65, NF‐κB p‐p65, IκBα, p‐IκBα, NLRP3, ASC, caspase‐1 and P2X7 were purchased from Cell Signaling Technology Inc (Beverly, MA). PU (purity, >98%) was purchased from Sigma‐Aldrich (St. Louis, USA). Myeloperoxidase (MPO) Adenosine triphosphate (ATP) and Glutathione (GSH) commercial kit was purchased from Nanjing Jiancheng Bioengineering Institute (Nanjing, China). Iron Saturation kits was obtained from Beckman Coulter Life Sciences (Brea, CA, USA). IL‐1β, IL‐6 and TNF‐α ELISA kits for cytokines were purchased from BioLegend (San Diego, USA).

### 2.3 Endometritis model administration and treatment

In total, 72 mice were divided into control group, PU group (100 mg/kg), *S. aureus* group (1 × 10^7^ CFU per 200 μL PBS) and PU (25, 50 mg/kg and 100 mg/kg, respectively) + *S. aureus* group. *S. aureus* was cultured in broth at 37°C. Resuspension *S. aureus* in 1 mL PBS and adjust the concentration to 1 × 10^7^ CFU/mL. As previously described, mice were anaesthetised, and 100 μL of the mixed *S. aureus* suspension was inoculated into the bilateral uterine horn of the mice using a syringe with a 30‐gauge blunt needle to induce endometritis.^24^ Once the model is successfully established, each group of mice was treated with PU. According to previous report,^25^ PU injection is dissolved in physiological saline. 25, 50 and 100 mg/kg PU was injected into the abdominal cavity once a day for 7 days, and the same amount of physiological saline was injected into the control group mice. Finally, mice were killed and the uterus from each mouse was collected and stored at −80°C until use.

### 2.4 Tissue iron contents

Iron contents were measured according to the manufacturer's instructions. Briefly, 0.1 g fresh tissue block was mixed with 0.9 mL extracting solution and centrifuged at 12,000× for 10 min and the supernatant were taken for later use. The supernatant (300 μL) was taken in a 1.5‐mL EP tube. The chromogenic solution (150 μL) was added, incubated at 37°C for 10 min, centrifuged at 12,000 × g for 10 min. The supernatant (300 μL) was taken into the well. The OD values of each well at 593 nm were measured on the enzyme‐linked immunosorbent assay.

### 2.5 Haematoxylin and eosin staining

After excision from the mice, the uterus tissue was fixed in 10% paraformaldehyde for 24 h at 25°C, then embedded with paraffin. Continuous 5 μm sections of the embedded tissue were prepare. Then, dewaxing treatment was performed and stained with haematoxylin and eosin, respectively. After dehydration, air‐dried sections were sealed with neutral resin. The routine morphological evaluation of the sections were performed using the optical microscope (Olympus, Tokyo, Japan).

### 2.6 Myeloperoxidase activity detection

The content of neutrophils in the uterus was determined using an MPO assay kit (Jiancheng Bioengineering Institute of Nanjing, Nanjing, China). In brief, 0.18 mL of the samples were placed into the centrifuge tubes and 0.02 mL of reagent 3, 0.2 mL of reagent 4 and 3 mL of chromogenic agent were added, respectively, and incubated at 37°C for 30 min. Then, 0.05 mL of reagent 7 was added and incubated at 60°C for 10 min. The enzymatic activity was detected with a spectrophotometer at 460 nm.

### 2.7 Enzyme‐linked immunosorbent assay (ELISA)

0.03 g of tissue was collected and homogenized with cold PBS in a 1:9 ratio in a tissue homogenizer. The levels of IL‐1β, IL‐6, TNF‐α, MDA, ATP and GSH were measured using the commercial kits according to the guidelines. Briefly, Capture Antibody was added to each well for 16 h, then 100 μL/well of samples was added to the wells. After that, 100 μL of Detection Antibody, Avidin‐HRP, Solution D, and Stop Solution was added to each well, respectively. Finally, the results were measured at 450 and 570 nm within 15 min.

### 2.8 Western blot analysis

0.05 g of the tissue was weighed, 300 uL of tissue lysate was added, and then the tissue in protein lysis buffer was homogenized, centrifuged at 12000 rpm at 4°C for 10 min and the total protein from the supernatant was extracted. Afterwards, the total protein content was determined using the BCA Protein Assay Kit (Thermo Fisher Scientific, USA). Protein was separated by 10% or 12% SDS–PAGE glue and transferred to the polyvinylidene fluoride (PVDF) membranes. PVDF membrane was sealed with 5% skim milk for 3 h, and incubated overnight with specific antibodies at 4°C. After washing the membrane three times with tris‐buffered saline with Tween (TBST), the membrane with the second antibody was incubated for 2 h, and then washed with TBST for three times. The protein bands were visualized using ECL kit with an Amersham Imager 600 (Cytiva, Marlborough, MA, U.S.A.). ImageJ computer software was used to analyse the density of the immunoreactive bands.

### 2.9 Statistical analysis

All experiment values are described as the means ± SEM of at least three separate experiments. Statistical significance was identified by using one‐way analysis of variance (ANOVA) and two tailed Student's t‐test. Graph Pad Prism software 5.0 (GraphPad Software, Inc., La Jolla, CA, USA) was used to analyse statistical significance, *p* < 0.05 was regarded as a statistically significant difference.

## 3 RESULTS

### 3.1 PU alleviates histopathological damage in endometritis induced by *S. aureus*

**FIGURE 1:**
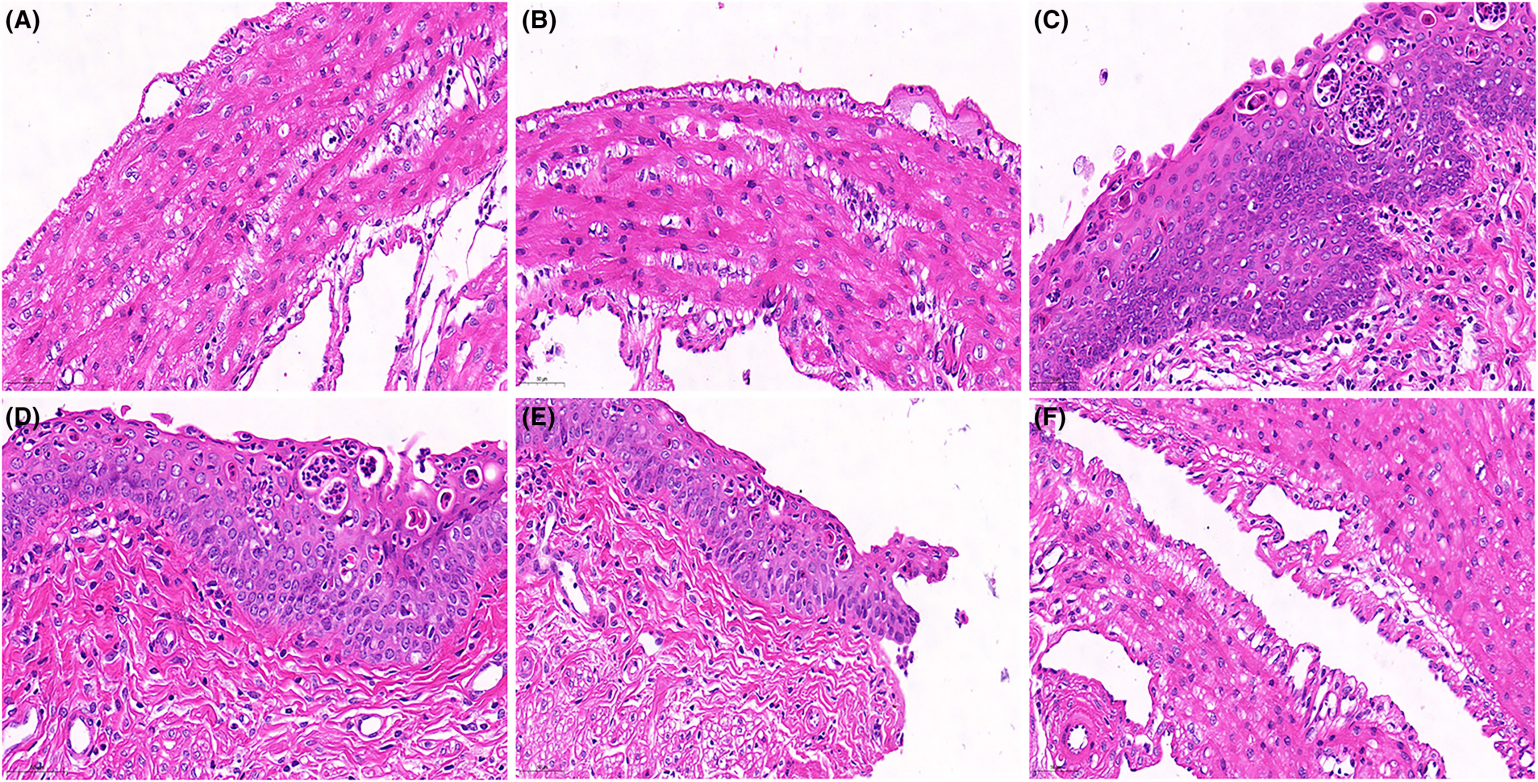
Puerarin (PU) alleviates histopathological damage in endometritis induced by *Staphylococcus aureus*. (A) Uterine tissues from control group, (B) treatment with PU alone, (C) *S. aureus* (1 × 10^7^ CFU per 100 μL PBS), (D) *S. aureus* + 25 mg/kg PU, (E) *S. aureus* + 50 mg/kg PU, (F) *S. aureus* + 100 mg/kg PU.

Detection of PU alleviating pathological damage induced by *S. aureus* in endometritis through HE staining, as shown in Figure 1. Compared with the control group, PU alone did not significantly change the morphology of mouse uterine tissue, while *S. aureus* caused significant uterine tissue oedema, severe infiltration of inflammatory cells and led to uterine structural disorder. The treatment of PU alleviated the uterine tissue damage induced by *S. aureus*, and as the concentration increased, the inflammatory damage decreased accordingly.

### 3.2 PU alleviates MPO in endometritis tissue induced by *S. aureus*

**FIGURE 2:**
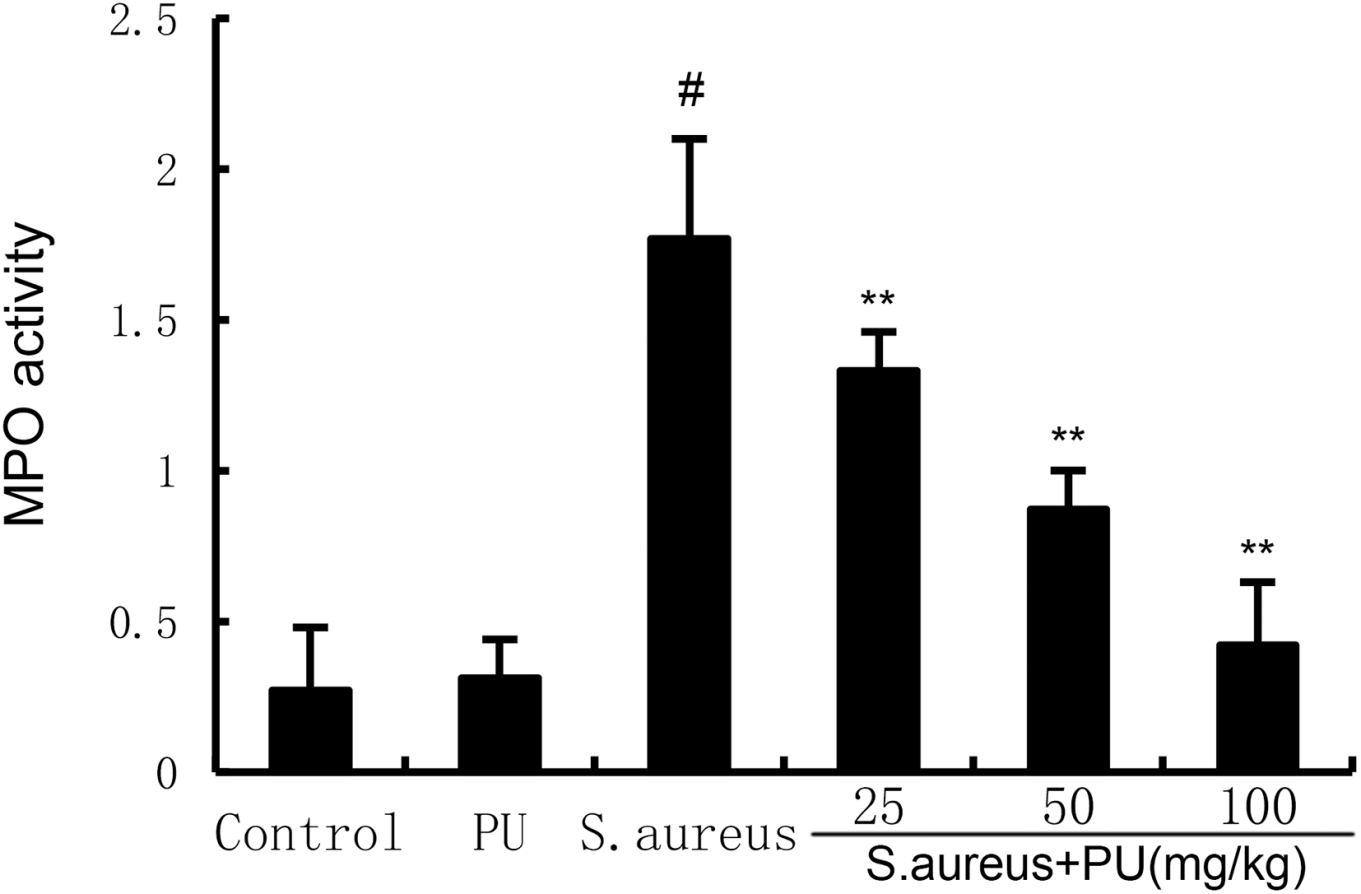
Puerarin (PU) alleviates MPO in endometritis tissue induced by *Staphylococcus aureus*. The values presented are the means ± SEM. #*p* < 0.05 is significantly different compared with control group. **p < 0.05 is significantly different compared with *S. aureus*.

The content of MPO in each neutrophil is certain; therefore, MPO can reflect the number of neutrophils.^26^ From Figure 2, it can be seen that compared with the control group, PU alone treatment did not affect the MPO activity of uterine tissue (*p* > 0.05), while *S. aureus* significantly promoted the activity of MPO (*p* < 0.05). Different concentrations of PU significantly alleviated the phenomenon of increased MPO activity induced by *S. aureus* (*p* < 0.05).

### 3.3 PU alleviates inflammatory response induced by *S. aureus*

**FIGURE 3:**
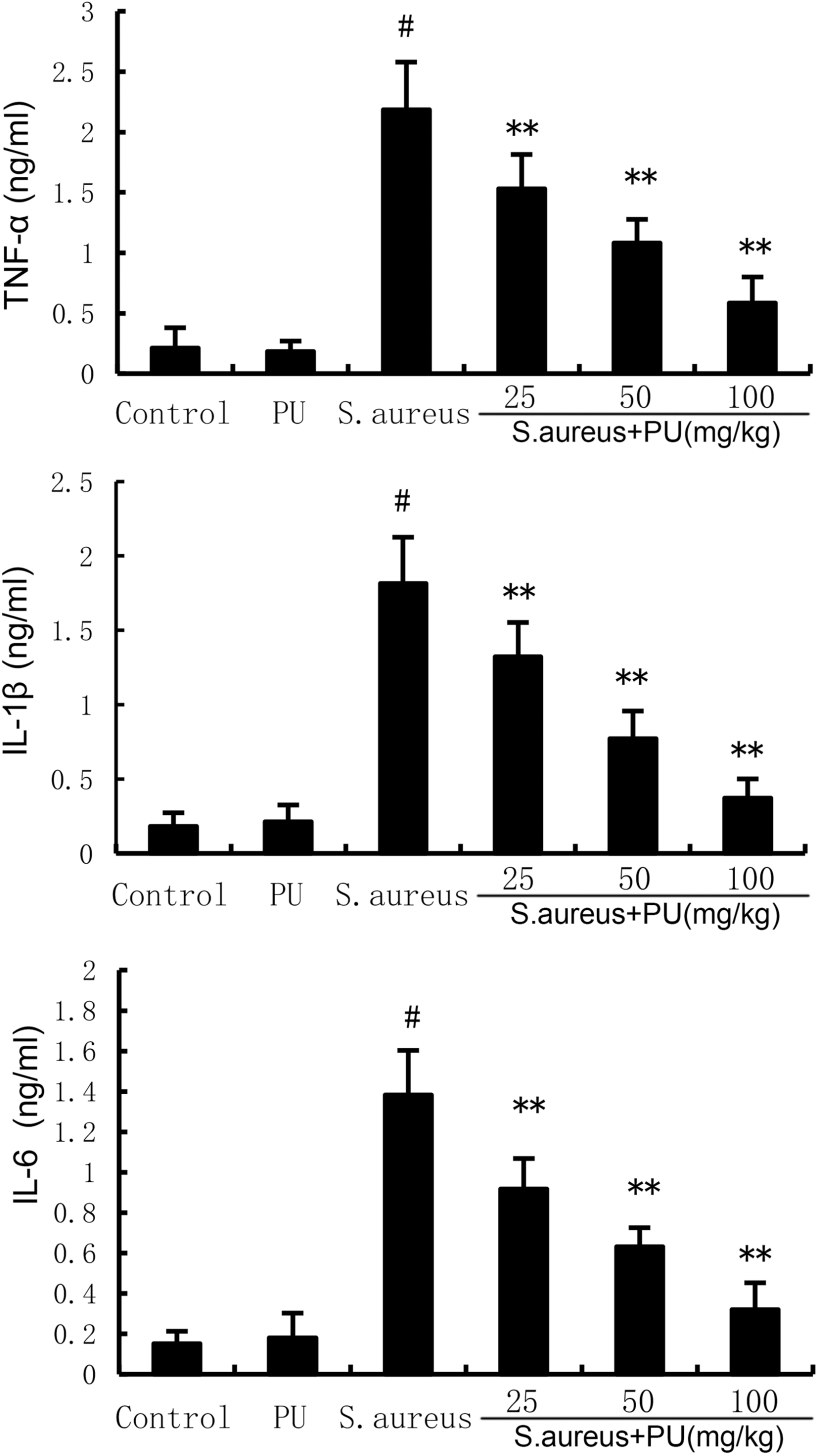
Puerarin (PU) alleviates inflammatory response induced by *Staphylococcus aureus*. The values presented are the means ± SEM. #*p* < 0.05 is significantly different compared with control group. **p < 0.05 is significantly different compared with *S. aureus*.

Figure 3 shows the level of pro‐inflammatory cytokines. TNF‐α, IL‐1 and IL‐6 are the main pro‐inflammatory cytokines, and their levels reflect the severity of endometritis. Compared with the control group, PU alone treatment did not affect the level of TNF‐α, IL‐1β and IL‐6 (*p* > 0.05), while *S. aureus* significantly promoted the levels of pro‐inflammatory cytokines (*p* < 0.05). Compared with *S. aureus*, different concentrations of PU significantly reduced the levels of inflammatory factors induced by *S. aureus* in a dose‐dependent manner (*p* < 0.05). It is speculated that PU can effectively treat *S. aureus*‐induced endometritis.

### 3.4 PU alleviates ferroptosis induced by *S. aureus*

**FIGURE 4:**
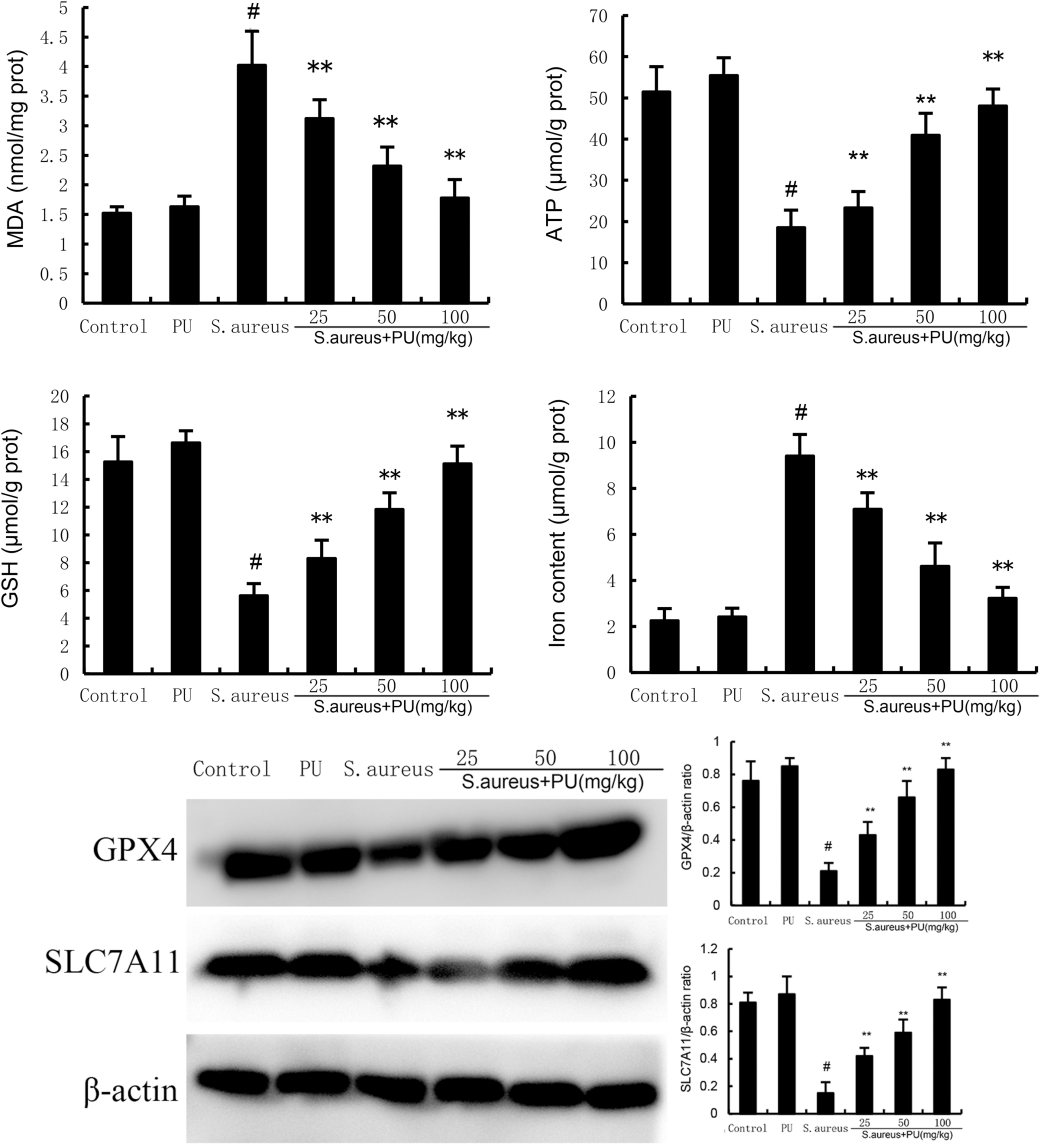
Puerarin (PU) alleviates ferroptosis induced by *Staphylococcus aureus*. The values presented are the means ± SEM. #*p* < 0.05 is significantly different compared with control group. **p < 0.05 is significantly different compared with *S. aureus*.

To analyse the effect on ferroptosis, we measured the levels of MDA, ATP, GSH and iron content in uterine tissues after treatment with *S. aureus* and PU. Compared with control group, PU did not affect the levels of MDA, ATP, GSH and iron content (*p* > 0.05), while *S. aureus* significantly increased the levels of MDA and iron content and decreased the levels of ATP and GSH (*p* < 0.05). In addition, *S. aureus* treatment significantly reduced the protein levels of GPX4 and SLC7A11 (*p* < 0.05), while PU reversed the effect of *S. aureus* on these indicators (*p* < 0.05). These results suggested that PU can alleviate ferroptosis of uterus induced by *S. aureus* (Figure 4).

### 3.5 PU inhibited the activation of the NF‐κB signalling pathway induced by *S. aureus*

**FIGURE 5:**
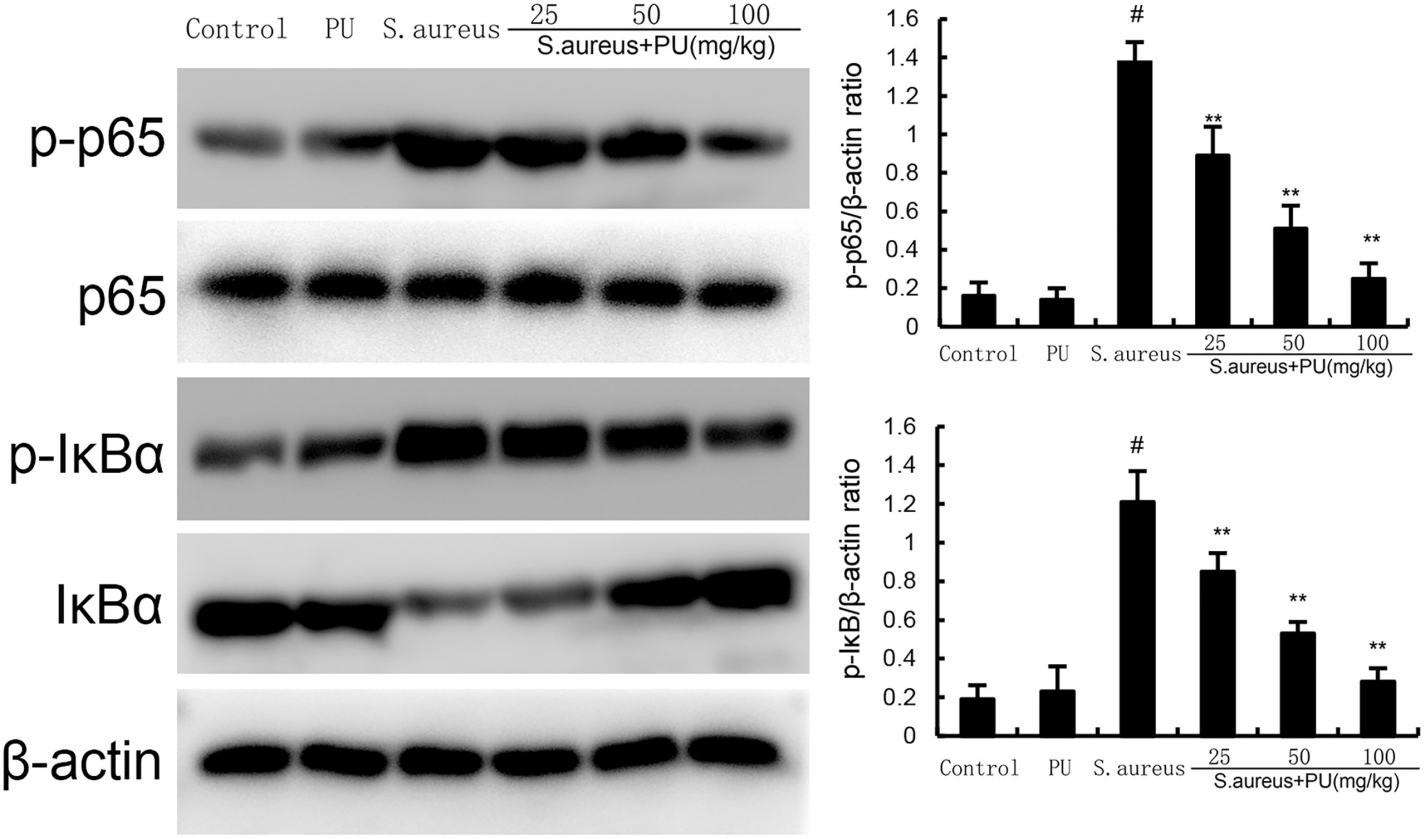
Puerarin (PU) inhibited the activation of the NF‐κB signalling pathway induced by *Staphylococcus aureus*. The values presented are the means ± SEM. #*p* < 0.05 is significantly different compared with control group. **p < 0.05 is significantly different compared with *S. aureus*.

We evaluated the effect of PU on the NF‐κB signalling pathway of endometritis induced by *S. aureus*. As shown in Figure 5, compared with control group, PU alone treatment did not affect the NF‐κB signalling pathway (*p* > 0.05), while *S. aureus* significantly promoted the expression of p‐p65 and p‐IκBα (*p* < 0.05). Compared with *S. aureus*, different concentrations of PU significantly reduced the expression of p‐p65 and p‐IκBα induced by *S. aureus* in a dose‐dependent manner (*p* < 0.05).

### 3.6 PU inhibited the expression of NLRP3 induced by *S. aureus*

**FIGURE 6:**
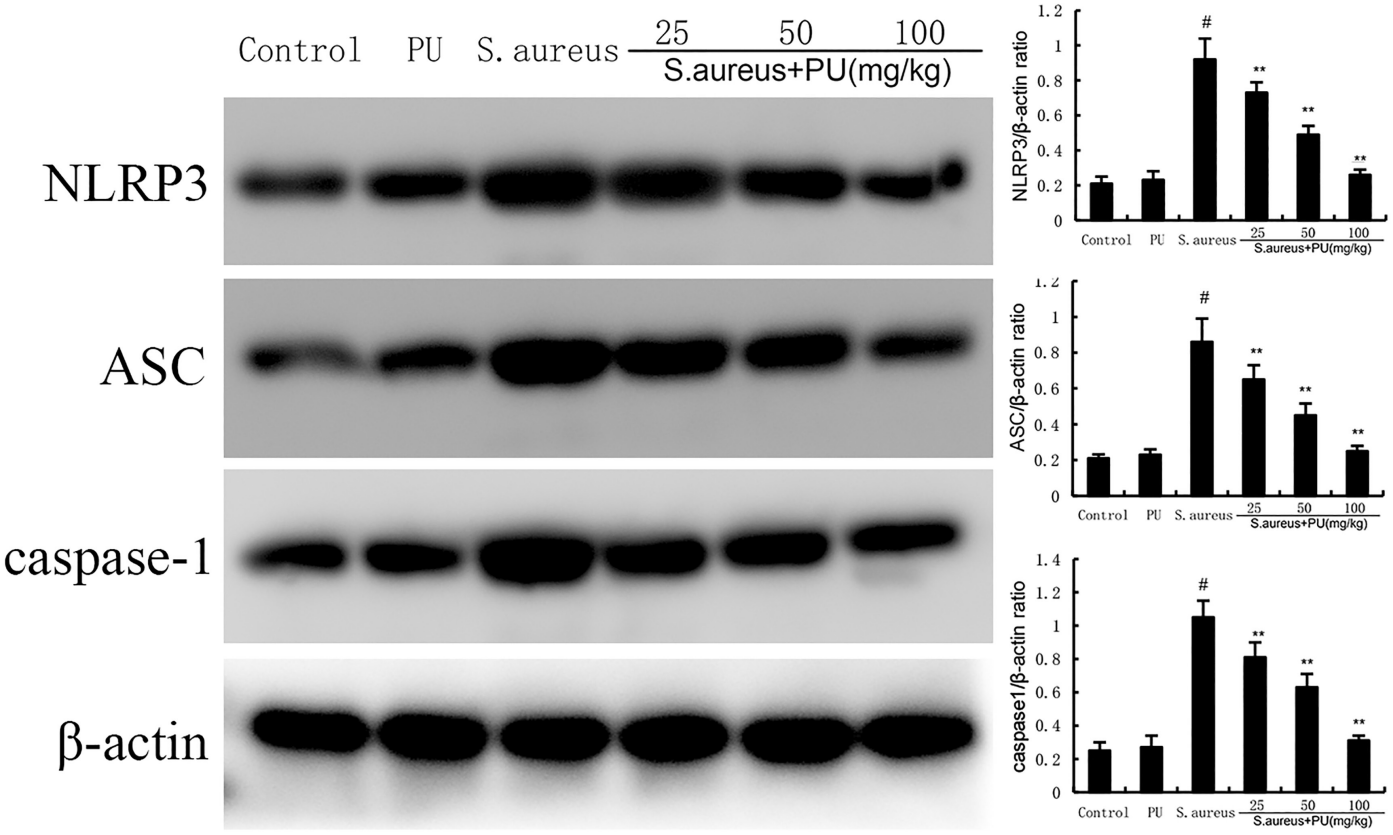
Puerarin (PU) inhibited the expression of NLRP3 induced by *Staphylococcus aureus*. The values presented are the means ± SEM. #*p* < 0.05 is significantly different compared with control group. **p < 0.05 is significantly different compared with *S. aureus*.

NLRP3 inflammasomes play an important role in inflammation, and apoptosis‐associated speck‐like protein (ASC) are important junction protein molecules in inflammasomes.^27^ As shown in Figure 6, compared with control group, *S. aureus* significantly increased the expression of NLRP3 and ASC (*p* < 0.05), while PU decreased the expression of NLRP3 and ASC compared with *S. aureus* group (*p* < 0.05).

### 3.7 PU inhibited the expression of P2X7 induced by *S. aureus*

**FIGURE 7:**
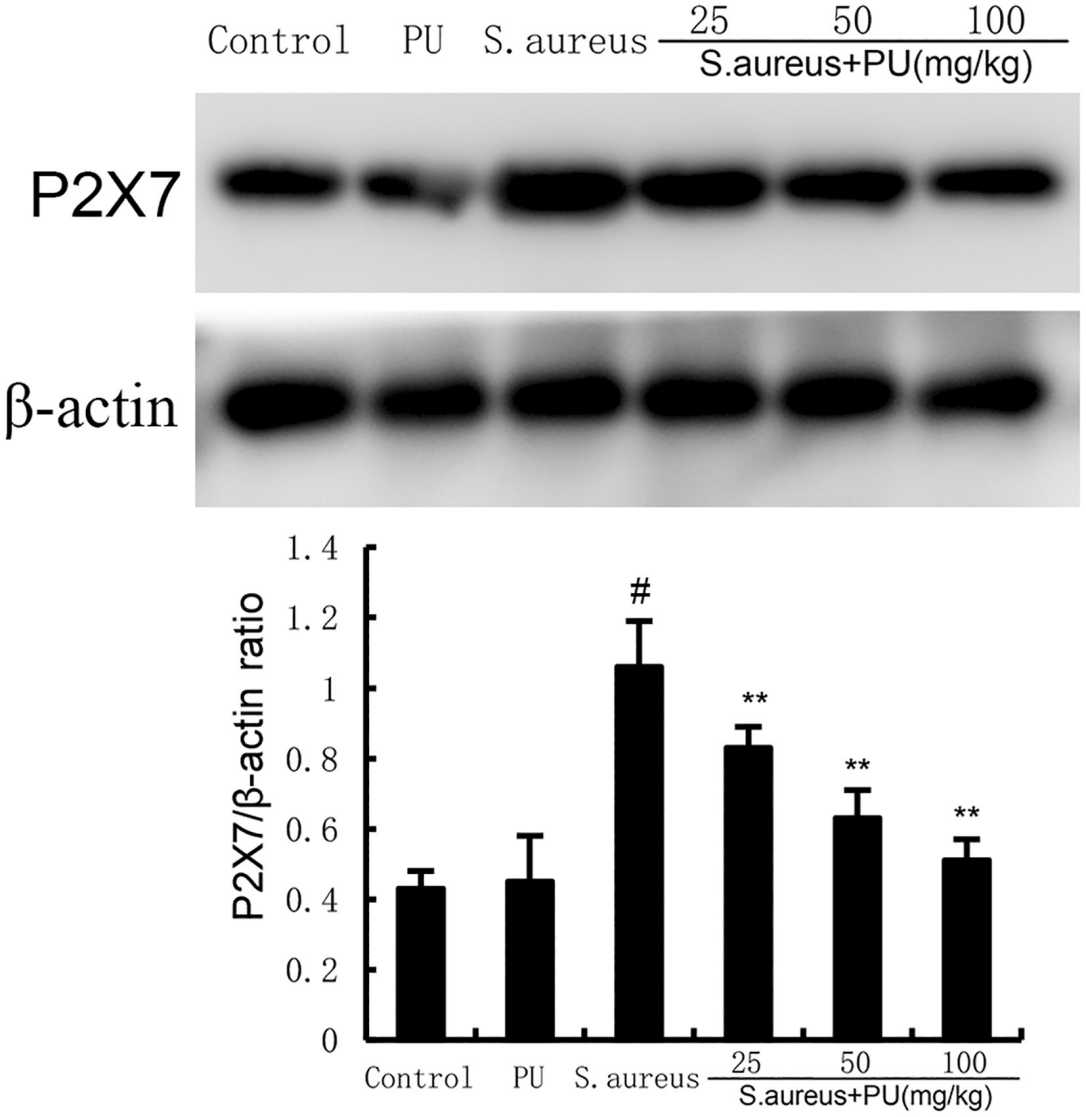
Puerarin (PU) inhibited the expression of P2X7 induced by *Staphylococcus aureus*. The values presented are the means ± SEM. #*p* < 0.05 is significantly different compared with control group. **p < 0.05 is significantly different compared with *S. aureus*.

Furthermore, we detected the expression of P2X7 in uterine tissue. It can be seen form Figure 7, compared with control group, *S. aureus* significantly increased the expression of P2X7 (*p* < 0.05), while PU reversed the effect of *S. aureus* on the expression of P2X7 in a dose‐dependent manner (*p* < 0.05). Indicating that PU may alleviate endometritis induced by *S. aureus* through the P2X7 signalling pathway.

## 4 DISCUSSION

In this study, we measured the protective effects of PU on *S. aureus*‐induced endometritis. Our study found that PU had beneficial role in protecting against endometritis. PU can alleviate endometritis by inhibiting ferroptosis via P2X7 receptor/NLRP3 signalling pathway. Therefore, PU has the potential to replace antibiotics in the treatment of endometritis.

Endometritis is a common inflammatory injury in the reproductive system.^4^ Bacterial infection is one of the most important factors leading to endometritis.^28^ *S. aureus* is the main bacterium in endometritis.^29^ The mouse endometritis model induced by intrauterine infusion of *S. aureus* has been widely used to explore the pathological mechanism of endometritis.^30^, ^31^ Therefore, this study used mouse model of endometritis induced by *S. aureus* for the experiment. Our study found that *S. aureus* could induce endometritis, which indicates that the mouse endometritis model induced by *S. aureus* is reliable.

At present, antibiotics are the main method for treating endometritis, but the use of antibiotics can increase bacterial resistance. Meanwhile, antibiotics not only kill harmful microbiota, but also beneficial microbiota.^32^ Therefore, it is necessary to find safe and effective drugs or methods to prevent and treat endometritis. PU is a flavonoid component isolated from *Pueraria lobata*, which has been clinically used for its protection against inflammation.^33^ PU can inhibit and alleviate LPS‐induced acute lung injury by inhibiting inflammatory response.^34^ In addition, PU may reduce LPS induced inflammation by activating the AMPK/SIRT1 signalling pathway, thereby inhibiting NLRP3 inflammasome‐mediated cell apoptosis.^35^ Our study results indicate that, PU reduces the increase of endometrial pro‐inflammatory factors such as TNF‐α, IL‐1β and IL‐6 induced by *S. aureus*. Inflammatory factors are involved in the development of endometritis. TNF‐α is responsible for the accumulation of leukocyte, participating in and promoting the inflammatory response.^36^ TNF‐α can exacerbate oxidative stress in the inflammatory site and participate in the formation of oedema, leading to further deterioration of inflammation.^37^ IL‐1β is one of the most pro‐inflammatory members of the IL‐1 family and can participate in the occurrence of various inflammatory diseases.^38^ In the early stages of inflammation, IL‐1β can promote the expression of endothelial cells and the chemotaxis of neutrophils. It is reported that IL‐1β can induce liver injury and inflammation in mice, and participate in systemic inflammation caused by asthma.^39^ It can be inferred that PU can effectively reduce *S. aureus*‐induced endometritis. NF‐κB is an important transcriptional regulatory factor in cells that typically binds to IκB and is in an inactive state. NF‐κB induces the expression of various genes through the activation of stimulating factors such as viruses, tumour necrosis factor, B‐cell activation factor, lymphotoxins, etc. and produces various cytokines to participate in the inflammatory response.^3^ Our results showed that PU could inhibit the expression of p‐p65 and p‐IκBα protein, which are related to inflammation. In summary, PU may inhibit the occurrence of inflammation through the NF‐κB signalling pathway.

Although these studies have shown that PU plays an important role in inflammatory response, the pathogenesis of PU in endometritis is currently unclear. Our study results found that PU can inhibit the increase in MDA and iron content caused by endometritis, and promote the content of ATP and GSH, indicating that PU can inhibit the occurrence of ferroptosis, which is similar to the previous study.^25^ More and more evidence suggest that the pathogenesis of endometritis is related to ferroptosis.^23^, ^40^ Ferroptosis is a nonapoptotic cell death pattern caused by iron dependent lipid peroxide accumulation.^41^ Ferroptosis is known to have complex regulatory pathways, which inevitably play important pathological roles in various diseases. Ferroptosis is accompanied by the release of pro‐inflammatory molecules such as TNF‐α and IL‐1β.^42^ Previous research has shown that IL‐1β induces activation of the P53‐XCT‐GSH axis in endothelial cells, leading to ferroptosis and endothelial dysfunction.^43^ It is consistent with our findings, which suggest that ferroptosis promotes an increase in IL‐1β levels in uterine tissue, indicating that IL‐1β is expected to become an important target for treating ferroptosis‐related inflammation. Ferroptosis can exacerbate the inflammatory response to varying degrees, and regulating ferroptosis is considered an effective intervention strategy for preventing and treating inflammatory diseases.^44^ In this study, we found that ferroptosis is involved in the development of endometritis, and PU significantly inhibits the reduction of expression of ferroptosis‐related proteins GPX4 and SLC7A11 induced by *S. aureus*. It can be speculated that PU can prevent *S. aureus*‐induced endometritis by inhibiting ferroptosis and inflammatory response. Whereas, the mechanism of anti‐ferroptosis effects of PU is still unknown.

NLRP3 inflammasomes have been shown to be involved in ferroptosis.^45^ Among inflammasomes, the NLRP3 inflammasome has been the most extensively studied and is involved in the process of innate immunity and adaptive immunity.^46^ When excessive accumulation of intracellular ferroptosis occurs, the cGAS‐STING pathway drives the activation of NLRP3 inflammasomes, further inducing oxidative stress, lipid peroxidation and ferroptosis.^47^ In addition, ferroptosis inhibitors can reduce the expression of NLRP3, IL‐1 and caspase‐1.^48^ The inducer of ferroptosis will activate NLRP1 and NLRP3 inflammasomes in cells, which will further promote the maturation of IL‐1 and promote the occurrence of inflammation.^49^ The P2X7 receptor is involved in regulating innate immunity mediated by the release of pro‐inflammatory cytokines such as IL‐1 and IL‐18.^50^ It is reported that after LPS or other bacterial endotoxins act on macrophages, the P2X7 receptor is activated, leading to the activation of NLRP3 inflammasome.^51^ Furthermore, the activation of P2X7 receptors can cause various biological reactions such as cell apoptosis, phagocytosis and pro‐inflammatory cytokine release.^52^ However, it is still unclear whether P2X7/NLRP3 signalling pathway can regulate endometritis. Our study results indicated that PU can reverse the increased expression of NLRP3, ASC, caspase‐1 and P2X7 proteins caused by endometritis, indicating that PU can inhibit the occurrence of endometritis through P2X7 receptor/NLRP3 signalling pathway.

## 5 CONCLUSION

In conclusion, the results of this study suggest that PU can alleviate endometritis induced by *S. aureus*. In addition, 100 mg/kg of PU has a better effect on alleviating endometritis. The mechanism of this protective role may be attributed to the attenuating ferroptosis via regulating P2X7 receptor/NLRP3 signalling pathway.

## AUTHOR CONTRIBUTIONS

**Chongshan Yuan:** Formal analysis (equal); investigation (lead); visualization (lead); writing – original draft (lead). **Li Liu:** Conceptualization (equal); data curation (lead); software (lead). **Ye Zhao:** Funding acquisition (lead); project administration (lead); supervision (lead); validation (lead). **Ke Wang:** Data curation (lead); project administration (equal); resources (lead); writing – review and editing (lead).

## CONFLICT OF INTEREST STATEMENT

The authors declare that they have no known competing financial interests or personal relationships.

## Data Availability

The data that support the findings of this study are available from the corresponding author upon reasonable request.
